# Preschool children from lower household incomes experience inequality in asthma treatment: findings from a Danish nationwide cohort study

**DOI:** 10.1093/eurpub/ckad187

**Published:** 2023-10-20

**Authors:** Camilla Klinge Renneberg, René Børge Korsgaard Brund, Signe Heuckendorff, Artika Gunaseelan, Lisbeth Venø Kruse, Kirsten Fonager

**Affiliations:** Department of Social Medicine, Aalborg University Hospital, Aalborg, Denmark; Department of Social Medicine, Aalborg University Hospital, Aalborg, Denmark; Department of Social Medicine, Aalborg University Hospital, Aalborg, Denmark; Psychiatry Region North Jutland, Aalborg, Denmark; Department of Social Medicine, Aalborg University Hospital, Aalborg, Denmark; Department of Paediatrics, Aalborg University Hospital, Aalborg, Denmark; Department of Social Medicine, Aalborg University Hospital, Aalborg, Denmark; Department of Clinical Medicine, Aalborg University, Aalborg, Denmark

## Abstract

**Background:**

The obligation to pay for asthma medication in a country with universal healthcare might lead to increased asthma exacerbations and inequitable healthcare access for children from low-income households. Thus, the aim of this study was to examine the association between household income and childhood/preschool asthma regarding hospitalization and medication receipt.

**Methods:**

In this nationwide register-based cohort study, we encompassed all 3-year-old children residing in Denmark, born between 2000 and 2014, along with their linked parents and followed them until their sixth birthday. Household income was divided into quartiles. Asthma was categorized in two mutually exclusive groups as either the redemption of two prescriptions for asthma medication or receiving a hospital diagnosis. We utilized Poisson regression to estimate the risk ratio (RR).

**Results:**

The analysis included 834 422 preschool children. The prevalence of asthma dependent on medication alone was 7.3%, while 1.8% of children necessitated asthma-related hospitalization. Income inequality was evident across all income quartiles. Notably, children from the lowest income quartile had a RR of 0.95 (95% CI: 0.92–0.98) of redeeming asthma medication and conversely a RR of 1.18 (95% CI: 1.14–1.23) of asthma-related hospitalization.

**Conclusions:**

Despite universal healthcare, income inequality has a dual impact on children from low-income households. They face a diminished risk of redeeming asthma medication and a higher susceptibility to asthma-related hospitalizations indicating an unequal access to healthcare. Prioritizing efforts to reduce childhood health inequalities is crucial. However, further research, particularly qualitative studies, is needed to better comprehend the underlying mechanisms to address the complexities of income inequality.

## Introduction

The pervasive problem of health inequality in child health, including asthma, is worldwide.^1–4^ Asthma, a chronic condition commonly diagnosed during childhood, poses a substantial public health burden and has been labelled the epidemic of the 21st century.^5^ Asthma affects ∼10% of children^6^ and the prevalence has been increasing.^5^ Asthma not only impacts the everyday life of the child, but it can also entail consequences on educational attainment and other health-related outcomes.^7^ Thus, identifying preventable exposures that lead to asthma and exacerbation seems highly important but has proved challenging.^8^ Moreover, advocating for health equity in childhood is crucial since disparities during this stage of life are not only unjust but also give rise to unequal health outcomes throughout one’s lifetime.^1^

It has been proposed that poverty might contribute to both the aetiology, exacerbation, recognition and also management of asthma.^3^ This income inequality in asthma among children is a phenomenon that has been observed in both countries with universal healthcare^9–13^ and countries without.^3^^,^^4^^,^^14^ However, some studies have also found no association between low-income and childhood asthma.^15–18^ The discrepancy between these studies results could be due to variations in study design, settings, small sample sizes and limitations associated with self-reported measures of childhood asthma as opposed to more objective measures. Studies using validated, objective measures of asthma repeatedly pool data by combining hospitalization and medication use. However, combining data on medication and hospitalization in a country, such as Denmark, may not be suitable for examining the association between household income and childhood asthma because the universal healthcare system covers hospitalization costs but not fully medication expenses.^19^ This may potentially cloud the results of the analysis. The fact that individuals must pay for medication, may limit access to medication for those with lower incomes resulting in unequal access to healthcare. To provide policymakers with the necessary information to accommodate appropriate responses to address the inequality in childhood asthma and exacerbation of such, it is highly relevant to investigate whether income serves as a barrier to adequate asthma treatment, and whether children from low-income families have a higher risk of hospitalization due to asthma.

## Aim

Thus, the aim of our study was to examine the association between household income and childhood/preschool asthma regarding hospitalization and medication receipt.

## Methods

### Design

This study was designed as a Danish nationwide register-based cohort study.

### Setting

Denmark has a tax-funded universal healthcare system that provides free access to hospitalization and visits to general practitioner (GP). GP’s serve as gatekeepers to both in- and outpatient hospital care among others and >99% of Danish residents are enlisted at a GP. To obtain asthma medication in Denmark, a GP provides a prescription that can solely be filled at Danish pharmacies. All redeemed medications are then registered on an individually level through the personal registration number, a unique identifier assigned all Danish residents. While patients are required to pay for their prescribed medications, subsequent reimbursement exists. These reimbursements increase as the medication expenses increase.^19^ For instance, the initial reimbursement kicks in if you spend over DKK 1045 on prescribed medication within a 1-year period, and at this point, the government automatically provides a 50% reimbursement. In excess of DKK 3795 the reimbursement is 85%. This applies to all Danish citizens and is not income related.

### Data sources

Data on children who were admitted to any Danish hospital due to asthma were obtained from the Danish National Patient Registry.^20^ Information on redemption of childhood asthma medication was obtained from The Danish National Prescription Registry.^21^ Data on household income and maternal education were retrieved from The Income Statistics Register^22^ and The Population Education Registry,^23^ respectively. Furthermore, The Danish Civil Registration System^24^ provided information on date of birth, country of origin, maternal age and cohabitation status. Information concerning maternal smoking during pregnancy was obtained from the Danish Medical Birth Registry.^25^ Data on maternal mental health conditions were retrieved from Danish National Patient Registry,^20^ The Danish National Prescription Registry^21^ and The Danish National Health Service Register.^26^ Statistics Denmark conducted anonymization of data and linkage between the registers on an individual level utilizing the personal registration number applied in all Danish public registers.^19^

### Study population

We included all children in Denmark born between 1 January 2000 and 31 December 2014 and their parents. Children were excluded if the child was not residing in Denmark at the child’s third birthday (baseline). Similarly, children were excluded if there was missing information on household income, maternal employment status, education, age of giving birth, country of origin or cohabitation status.

### Variables

#### Exposure

The exposure of interest was defined as household income. The household was defined as consisting of the primary caregiver, their partner and any child of either adult under 25 years of age residing at the same address.^27^ At the child’s age of 2 years, the primary caregiver was determined as the parent with whom the child’s official address is registered. In Denmark, this is typically the mother if the parents are not living together, unless the father has full custody. Income was measured as the household’s equivalized disposable annual income and was determined by subtracting taxes and interest expenses (e.g. real estate interest) from the total household income. The household income was then divided by the equivalent size of the household using the modified OECD equivalence scale. This scale assigns a weight of 1.0 to the first adult, 0.5 to any other person aged 14 years or above, and 0.3 to each child under the age of 14 years. The entire calculation of household income was performed by Statistics Denmark, and it allows for comparison of household incomes independent of family size and age composition.^22^

Household income was categorized into four quartiles. The lowest quartile was further dichotomized based on maternal employment status, distinguishing between students (*Q*_1e_) and non-students (*Q*_1_).

To address the impact of factors, such as general wage increases and inflation on income, which can hinder income comparisons over time, we used calendar year-specific quartiles of the base income to adjust for these changes in household income.

Annual household income was extracted in the year prior to baseline.

#### Outcome

The outcome of interest was asthma, categorized into either medication or hospitalization. These two were mutually exclusive. Children categorized into medication was defined as no asthma*-*related hospital contact and at least two redeemed prescriptions of either inhaled long-acting b2-agonist (ATC R03AC), inhaled corticosteroids (ATC R03BA) fixed-dose combinations of inhaled b2-agonists, corticosteroids (ATC R03AK) and/or leukotriene-receptor antagonist (ATC R03DC).

In accordance with the International Classification of Diseases 10 (ICD-10) children with a hospital diagnosis of asthma (ICD-10: J45) or status asthmaticus (ICD-10: J46) were classified into hospitalization*.* Both in- and outpatient diagnosis were considered.

Asthma medication or hospitalization cases were identified for children between the ages of 3 and 6 years, due to onset of asthma typically occurring during the preschool years.^28^ At this stage, children are highly dependent on their parents to manage their asthma treatment. Children below 3 years of age were not considered, as they often experience lower respiratory tract infections during this period, possibly due to smaller airways, and furthermore do not have higher risk of future asthma.^29^ Therefore, asthma medication prescriptions previous to the age of 3 are more likely a result of lower respiratory tract infections rather than asthma.

#### Covariates

Through a review of the literature, potential confounding factors that could influence the association between household income and childhood asthma were identified. Subsequently, a directed acyclic graph (DAG) was then constructed to guide the analysis (see Supplementary figure S1).

As the primary caregiver most often is the mother, and she usually takes primary responsibility for the upbringing and care of the child,^30^ it is more likely that she is responsible for purchasing and administering the asthma medication. Furthermore, in Denmark, it is possible to be registered without specifying a father but not without specifying a mother. Therefore, all covariates were obtained for the mother.

Maternal mental health conditions were classified in four mutually exclusive severity groups: no, minor, moderate and severe. Minor mental health conditions were defined as those managed in primary healthcare or ≥2 redeemed prescriptions of antidepressants or benzodiazepines. Moderate mental health conditions were identified by contact with a private psychiatrist or an ICD-10: F00-F99 diagnosis registered at a Danish psychiatric hospital, except those defining the severe mental health condition group such as schizophrenia (ICD-10: F20-F22). For a more detailed description of the categorization of mental health conditions, please refer to Supplementary table S1. Since mental health conditions can be persistent or recurrent,^31^ we examined all contacts, conditions and medications related to mental health within a period of 5 years before baseline.

Based on the International Standard Classification of Education (ISCED), we categorized length of maternal education into three groups: short (primary school education), intermediate (secondary or vocational education) and long (short-cycle tertiary education or above), corresponding to ISCED levels 0–2, 3–4 and 5–8, respectively. For more information regarding this categorization, see Supplementary table S2. Maternal education was extracted at baseline.

We categorized employment status of mothers into three categories: wage earners, recipients of social benefits and students. Wage earners included those with a main income source from paid employment, including sick leave or unemployment benefits. Recipients of social benefits included mothers whose main income source was social welfare benefits, excluding unemployed or students.

Cohabitation status was categorized into living alone, cohabitant with father or cohabitant with another partner at baseline. Maternal age was measured at the time of giving birth to the child.

Maternal country of origin was categorized into Nordic countries, Western Europe and other. Maternal smoking during pregnancy was dichotomized as either yes or no, based on self-reported information reported to the midwife during the first trimester.

### Follow-up

Children were followed from their third birthday (baseline) until their sixth birthday, death, emigration or end of follow-up on 15 March 2018, whichever came first.

### Statistics

Poisson regression models was used to estimate the risk ratios (RRs) and its corresponding 95% confidence interval (95% CI). Analyses were based on complete case data and children from the highest income quartile was considered the reference group.

The analyses were adjusted for maternal mental health conditions, education, employment status, cohabitation status and age. Maternal age at giving birth was adjusted using restricted cubic splines with three knots. Furthermore, as missing information on household income showed a strong association with maternal country of origin, we incorporated this variable as a covariate in our analysis. This adjustment aimed to mitigate potential selection bias that could arise from the missing data.^32^

As we limited our analysis to maternal covariates, we performed a sensitivity analysis in which we incorporated the corresponding paternal covariates.

### Ethics

All methods were conducted in accordance with relevant regulations and guidelines, including the 1975 Declaration of Helsinki. The project complies with Danish laws and European Union directives on personal data protection. Approval for data usage and the study was obtained from the Danish Data Protection Agency (Region North of Denmark—Approval number 2019–2078). The study used existing administrative national registries, and data anonymity was ensured. The study only includes aggregated results without personal data. As a register-based study under Danish law, ethical committee approval and written consent are not required.^33^

## Results

**Figure 1:**
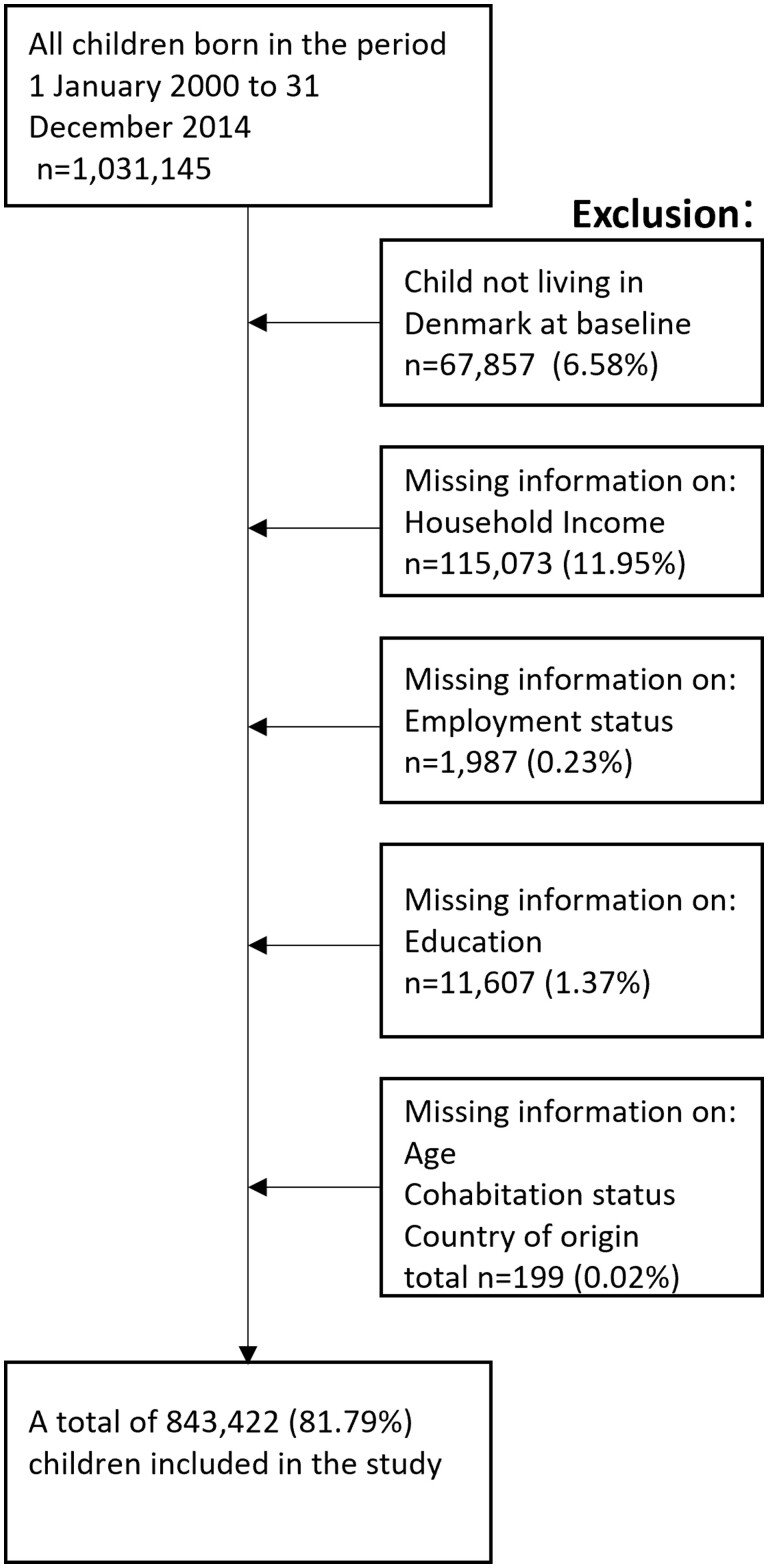
Flowchart of the inclusion, exclusion and final study population. We planned to exclude children from the analysis if both parents had a missing personal registration number. However, none of the children fulfilled this criterion

A total of 834 422 children were included in this study (figure 1).

The baseline characteristics are displayed in table 1. Among mothers with the lowest household income (*Q*_1_), it was observed that they had a shorter educational level compared to those in higher income quartiles. Additionally, a higher proportion of mothers in the lowest income quartile were recipients of social benefits, resided alone and had mental health conditions. Furthermore, a larger percentage of mothers from this group originated from countries outside of the Nordic or Western European regions. Also, the prevalence of smoking during pregnancy was found to be higher among mothers in the lowest household income quartile when compared to those in the highest quartile (*Q*_4_).

**Table 1:** Baseline characteristics by annual household income quartiles

**Household income quartiles** ^a^	*Q*1	*Q*1e	*Q*2	*Q*3	*Q*4
*n* (%)	184 010 (22.1)	24 595 (2.9)	208 606 (25.0)	208 605 (25.0)	208 606 (25.0)
Maternal education, *n* (%)					
Short	73 686 (40.0)	4604 (18.7)	29 838 (14.3)	13 187 (6.3)	6226 (3.0)
Intermediate	70 655 (38.4)	10 081 (41.0)	96 234 (46.1)	79 379 (38.1)	54 656 (26.2)
Long	39 669 (21.6)	9910 (40.3)	82 534 (39.6)	116 039 (55.6)	147 724 (70.8)
Maternal employment status, *n* (%)					
Social benefits	73 387 (39.9)	0 (0.0)	12 055 (5.8)	4074 (2.0)	3337 (1.6)
Students	0 (0.0)	24 595 (100.0)	11 267 (5.4)	4538 (2.2)	2346 (1.1)
Wage earners	110 623 (60.1)	0 (0.0)	185 284 (88.8)	199 993 (95.9)	202 923 (97.3)
Maternal age at birth, median	29.6 (25.8; 33.7)	26.3 (23.7; 29.3)	30.1 (27.1; 33.5)	31.0 (28.3; 34.0)	32.4 (29.9; 35.3)
Maternal cohabitation status, *n* (%)					
Cohabitant with father	149 010 (81.0)	20 346 (82.7)	200 688 (96.2)	206 330 (98.9)	207 341 (99.4)
Cohabitant with another partner	5750 (3.1)	377 (1.5)	728 (0.3)	278 (0.1)	214 (0.1)
Living alone	29 250 (15.9)	3872 (15.7)	7190 (3.4)	1997 (1.0)	1051 (0.5)
Maternal country of origin, *n* (%)					
Nordic	119 216 (64.8)	21 235 (86.3)	188 017 (90.1)	196 958 (94.4)	197 346 (94.6)
Western Europe	2929 (1.6)	242 (1.0)	1959 (0.9)	1903 (0.9)	3237 (1.6)
Other	61 865 (33.6)	3118 (12.7)	18 630 (8.9)	9744 (4.7)	8023 (3.8)
Maternal mental health condition, *n* (%)					
No	129 680 (70.5)	16 885 (68.7)	154 917 (74.3)	162 846 (78.1)	169 300 (81.2)
Minor	30 772 (16.7)	4833 (19.7)	37 828 (18.1)	34 714 (16.6)	30 522 (14.6)
Moderate	20 746 (11.3)	2626 (10.7)	14 361 (6.9)	10 268 (4.9)	8158 (3.9)
Severe	2812 (1.5)	251 (1.0)	1500 (0.7)	777 (0.4)	626 (0.3)
Maternal smoking during pregnancy, *n* (%)					
No	124 125 (67.5)	19 716 (80.2)	169 004 (81.0)	181 439 (87.0)	187 983 (90.1)
Yes	33 192 (18.0)	2907 (11.8)	27 488 (13.2)	17 267 (8.3)	9225 (4.4)
Missing information	26 693 (14.5)	1972 (8.0)	12 114 (5.8)	9899 (4.7)	11 398 (5.5)

a *Q*1 = lowest 25% quartile, *Q*1e=lowest 25% quartile based on maternal employment status, *Q*2, 25–50% quartile, *Q*3 = 50–75% quartile and *Q*4 = highest 75% quartile.

**Figure 2:**
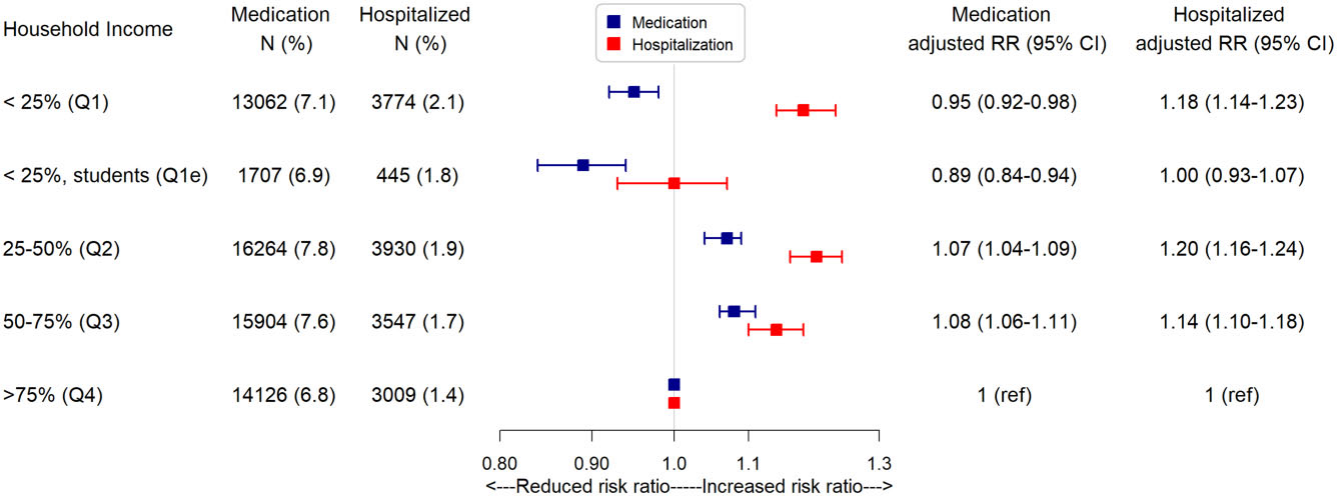
Adjusted RR for preschool asthma medication redemption and asthma-related hospitalizations based on household income quartiles. Analysis adjusted for maternal mental health conditions, maternal education, employment status, cohabitation status, maternal age and maternal country of origin. 95% CI: 95% confidence interval

In general, the prevalence of asthma solely reliant on medication was found to be 7.3%. Moreover, it was observed that 1.8% of children in the study population required hospitalization due to asthma. Children from the lowest household income quartile (*Q*_1_) experienced a 5% lower risk of receiving asthma medication (RR 0.95, 95% CI: 0.92–0.98). Conversely, these children faced an 18% higher risk of asthma-related hospitalization (RR 1.18, 95% CI: 1.14–1.23) compared to children from the highest household income quartile (*Q*_4_). Please refer to figure 2.

The subgroup of children from the lowest household income quartile whose mothers were students (*Q*1e), had an 11% lower risk of receiving asthma medication (RR 0.89, 95% CI: 0.84–0.94). However, no association was found between this group and asthma-related hospitalization (RR 1.00, 95% CI: 0.96–1.07).

Compared to children from the highest household income quartile (*Q*_4_) children from the second (*Q*_2_) and third (*Q*_3_) income quartile had a 7% (RR 1.07, 95% CI: 1.04–1.09) and an 8% (RR 1.08, 95% CI: 1.06–1.11) higher risk of redeemed asthma medication, respectively. Regarding hospitalization due to asthma, children from *Q*_2_ had a 20% (RR 1.20, 95% CI: 1.16–1.24) higher risk and children from *Q*_3_ had a 14% (RR 1.14, 95% CI: 1.10–1.18) higher risk. The crude estimates are shown in Supplementary tables S3 and S4.

Our sensitivity analysis, which incorporates paternal covariates, is presented in Supplementary tables S5 and S6. The results remained largely consistent with the main analysis, which included only maternal covariates, and the overall interpretation remained unchanged.

## Discussion

In this comprehensive nationwide cohort study, we disclose an income inequality in childhood asthma already among pre-schoolers. The findings demonstrate a divergence in the pattern of inequality concerning the risk of asthma when considering either redemption of asthma medication alone or hospitalization due to asthma. Notably, children from families with the lowest household income exhibited a lower risk of receiving asthma medication, while simultaneously facing a higher risk of asthma-related in- and out-hospitalization in comparison to children from the highest household income quartile. These findings indicate a dual nature of the income inequality, with observations in two opposed directions.

The overall income inequality observed in this study, as well as others,^9–13^ persists even within a nation that offers universal healthcare. However, to our knowledge, this is the first study to explore the association between household income and childhood/preschool asthma in terms of hospitalization and redeemed medication.

Underlying mechanisms explaining some of the dual nature of the income inequality found in this study could be that families with the lowest incomes, hence a lower socioeconomic position, may face challenges when it comes to accessing healthcare. These parents may delay seeking care from their GP or lack the necessary resources to do so effectively.^34^ Furthermore, it is known that GPs in Denmark are less inclined to establish their practices in sparsely populated areas, which tend to have a higher concentration of low-income families. Thus, these families may lack a stable relationship with their GP who might frequently change. Additionally, the requirement to pay for medications introduces a potential disparity in access to essential treatments. Low-income families may be disproportionately affected, as they might be forced to make difficult choices between purchasing medications or meeting other basic needs. Also, one study has observed that children aged 1–4 years from low-income families are found to receive later diagnosis of asthma compared to their counterparts from high-income families.^35^

It is well-established that poor adherence to asthma medication can elevate the risk of asthma exacerbations and subsequent hospitalizations,^36^ additionally, socially disadvantaged families tend to have less control over their children’s asthma.^37^ This poor adherence and compliance to treatment may be linked to lower health literacy, limited access to healthcare, alongside their limited resources to afford the medication. However, children from low-income households, whose mothers were students, showed no difference in the risk of asthma-related hospitalization compared to children from high-income households. This suggests that the higher risk of preschool asthma-related hospitalization among low-income children in this study may more likely be driven by limited access to healthcare, including appropriate treatment and asthma management programmes, as well as a lack of health literacy. Thus, household income alone may not fully account for this higher risk.

### Strengths and limitations

This study comprises numerous strengths as it uses high-quality and valid data from the national Danish registers, which provides comprehensive coverage of the entire population.^33^ This allowed for a nationwide large cohort study and enabled the opportunity to divide the lowest household income group into two separate groups, distinguishing recipients of social welfare benefits (*Q*_1_) from those with low income due to being students (*Q*_1e_). This division takes into consideration potential variations in resources beyond income creating the low-income group more homogeneous.

Furthermore, the study’s outcome of interest was based on validated measures of asthma, ensuring the reliability and accuracy of the findings when utilizing register-based data.^38^ Another strength of this study is the rigorous adjustment for numerous psychosocial maternal vulnerabilities, guided by our DAG, which has reduced confounding of the impact of household income on preschool asthma. This study also has some limitations, such as inherent limitations in its observational design based on registries. These limitations include restricted data availability, encompassing only a predetermined set of variables. Additionally, the cohort relies on redeemed prescription data, which may fail to identify children who have in fact received a prescription from their own GP but have not redeemed it. Furthermore, the reliance on prescription data poses challenges in evaluating essential aspects of asthma care, such as inhaler technique.

Furthermore, it is common to encounter missing data related to the household’s equivalized disposable annual income, which is calculated and obtained through Statistics Denmark. To address the potential selection bias resulting from excluding children with missing information on household income, we adjusted for maternal country of origin as this was highly associated with the missing data.

The causal link between second-hand smoking and childhood asthma is widely acknowledged and supported by extensive research.^39^ Furthermore, individuals from low-income backgrounds tend to have higher smoking rates compared to those with higher income levels.^40^ In our analysis, we made efforts to account for the impact of second-hand smoking by adjusting for other covariates that exhibit strong correlations with smoking, as illustrated in our DAG presented in Supplementary figure S1. Nevertheless, the absence of information on second-hand smoking leaves potential for unadjusted (residual) confounding, potentially introducing unaccounted biases in our analysis. While it is plausible that second-hand smoking may contribute to the higher rate of hospitalizations among children from low-income households, it does not provide a satisfactory explanation of why these children redeem less prescribed asthma medication. Hence, smoking cannot explain the dual nature of the inequality observed in this study.

### Implications of findings

The presence of the dual nature of inequality in asthma among preschool children from lower household incomes emphasizes a significant potential for improvement. Even within a society that provides universal healthcare our findings indicate an inequality in healthcare access.

Instead, our findings should serve as a strong motivation and provide support for governmental efforts to prioritize the reduction of health inequalities during childhood through the implementation of targeted health policies. By implementing targeted policies and interventions that address appropriate asthma treatment, adherence to treatment and ensure equal access to healthcare, public health benefits could be achieved. These interventions have the potential to reduce the burden of preventable hospital admissions and the associated excessive costs. However, it is crucial to acknowledge the complexity of the potential underlying mechanisms, which, this study by design is unable to address. Thus, more research is essential to explore this inequality, with a particular need for qualitative research to better understand the underlying mechanism that require attention.

## Conclusion

Despite the presence of universal healthcare, income inequality manifests in a dual nature when it comes to children from low-income households. They face a diminished risk of redeeming asthma medication and a higher susceptibility to asthma-related hospitalizations compared to their counterparts from high-income households indicating an unequal access to healthcare. It is crucial to prioritize efforts to reduce health inequalities during childhood. However, more research is needed, specifically to explore the complexities of income inequality. Conducting qualitative research would be particularly valuable to better understand the underlying mechanisms that require attention.

## Supplementary Material

ckad187_Supplementary_Data

## Data Availability

The data supporting the findings of this study can be accessed through Statistics Denmark. However, it is important to note that certain restrictions apply to its availability, and thus, the data are not publicly accessible. If you have any inquiries or requests related to these data, kindly direct them to the corresponding author, Camilla Klinge Renneberg.
