# Supplementary material for: Preschool children from lower household incomes experience inequality in asthma treatment: findings from a Danish nationwide cohort study
Source: Eur J Public Health. 2023 Oct 20;34(1):85–90. doi: 10.1093/eurpub/ckad187 (PMC10843934; doi:10.1093/eurpub/ckad187)
Supplement: ckad187_Supplementary_Data [file ckad187_supplementary_data.pdf]

European Journal of Public Health

Preschool children from lower household incomes experience inequality in asthma treatment: findings from a Danish nationwide cohort study

- Additional file 1 - Supplementary tables

Authors

Camilla Klinge Renneberg[1]*, ORCID: 0000-0002-8588-7637

René Børge Korsgaard Brund[1], ORCID: 0000-0002-7246-4811

Signe Heuckendorff[1,2], ORCID: 0000-0002-4074-064X

Artika Gunaseelan[1]

Lisbeth Venø Kruse[3], ORCID: 0009-0007-1192-9201

Kirsten Fonager[1,4], ORCID: 0000-0001-9641-992X

Affiliations

1. Department of Social Medicine, Aalborg University Hospital, Aalborg, Denmark
2. Psychiatry Region North Jutland, Aalborg, Denmark
3. Department of Paediatrics, Aalborg University Hospital, Aalborg, Denmark
4. Department of Clinical Medicine, Aalborg University, Aalborg, Denmark

*Corresponding author: Camilla Klinge Renneberg ca-klinge@live.dk

Supplementary Table s1: Definition of the four mutually exclusive severity groups of maternal mental health conditions

**Exposure of mental health conditions**	**Definition** (measured within the five years prior to baseline)
**Minor mental health conditions***	**At least one of the criteria fulfilled:**<br>**Medication**[a]- *At least two redeemed prescriptions of:*<br>- Anxiolytic medication (ATC: N03AE, N05BA, N05CD, N05CF)<br>- Antidepressant medication (ATC: N06AB, N06AX)<br>**Services at general practitioners** (GP)[b]<br>- At least two sessions of 'talk therapy'<br>- At least two psychometric tests<br>**Other services**[b]<br>- At least one contact to a private psychologist<br>**Additional criteria** *(all fulfilled)*<br>- No contacts or diagnoses from psychiatric hospital<br>- No records of contact to a private psychiatrist
**Moderate mental health conditions**	**At least one of the criteria fulfilled:**<br>**Psychiatric hospital**[c]<br>- Psychiatric diagnosis (ICD-10: Mental and behavioural disorders F00-99) registered at psychiatric hospital (both in- and outpatient contacts) - excluding the diagnoses defining the following severity group of severe mental health conditions.<br>**Other services**[b]<br>- At least one contact to a private psychiatrist
**Severe mental health conditions**	**At least one of the criteria fulfilled:**<br>**Psychiatric hospital**[c]<br>- Both in- and outpatient contact with a registered diagnosis of either schizophrenia (ICD-10: F20-22) or bipolar disease (ICD-10: F30-31)<br>- Inpatient contact with a registered diagnosis of either unipolar depression (ICD-10: F32-34) or emotionally unstable personality disorder (ICD-10: F60.3)
**No mental health conditions**	**None of the above**

[*] The majority of minor mental health conditions are treated by general practitioners or psychologists in the primary healthcare sector and therefore not registered with a diagnosis from any psychiatric hospital in the Danish national registers.

a *Data on medication was obtained from The Danish National Prescription Registry.*[1]

b *Data from GP, private psychologist and psychiatrist was obtained from The Danish National Health Service Register.*[2]

c *Psychiatric diagnosis registered at the psychiatric hospital was obtained from The Danish National Patient Register.*[3]

Supplementary Table s2: Definition of maternal education

EDUCATIONAL LEVEL	REGISTRY[4]	DESCRIPTION
**Short education**	Statistics Denmark's registry on education (UDDA)	**ISCED Levels 0-2:** Early childhood education, primary education and lower secondary education corresponding to 1-10 years of education
**Intermediate education**	Statistics Denmark's registry on education (UDDA)	**ISCED Levels 3-4:** General upper secondary education, high school programs, vocational upper secondary education, vocational training, and education corresponding to approximately 10-12 years of education
**Long education**	Statistics Denmark's registry on education (UDDA)	**ISCED Levels 5-8:** Short, medium-length or long length higher education, first, second or third cycle programs, tertiary education, bachelor or equivalent, master's or equivalent, Doctoral, PhD programs or equivalent corresponding to more than approximately 12 years of education

Maternal education was extracted at baseline.

Supplementary Figure s1: Directed Acyclic Graph - DAG

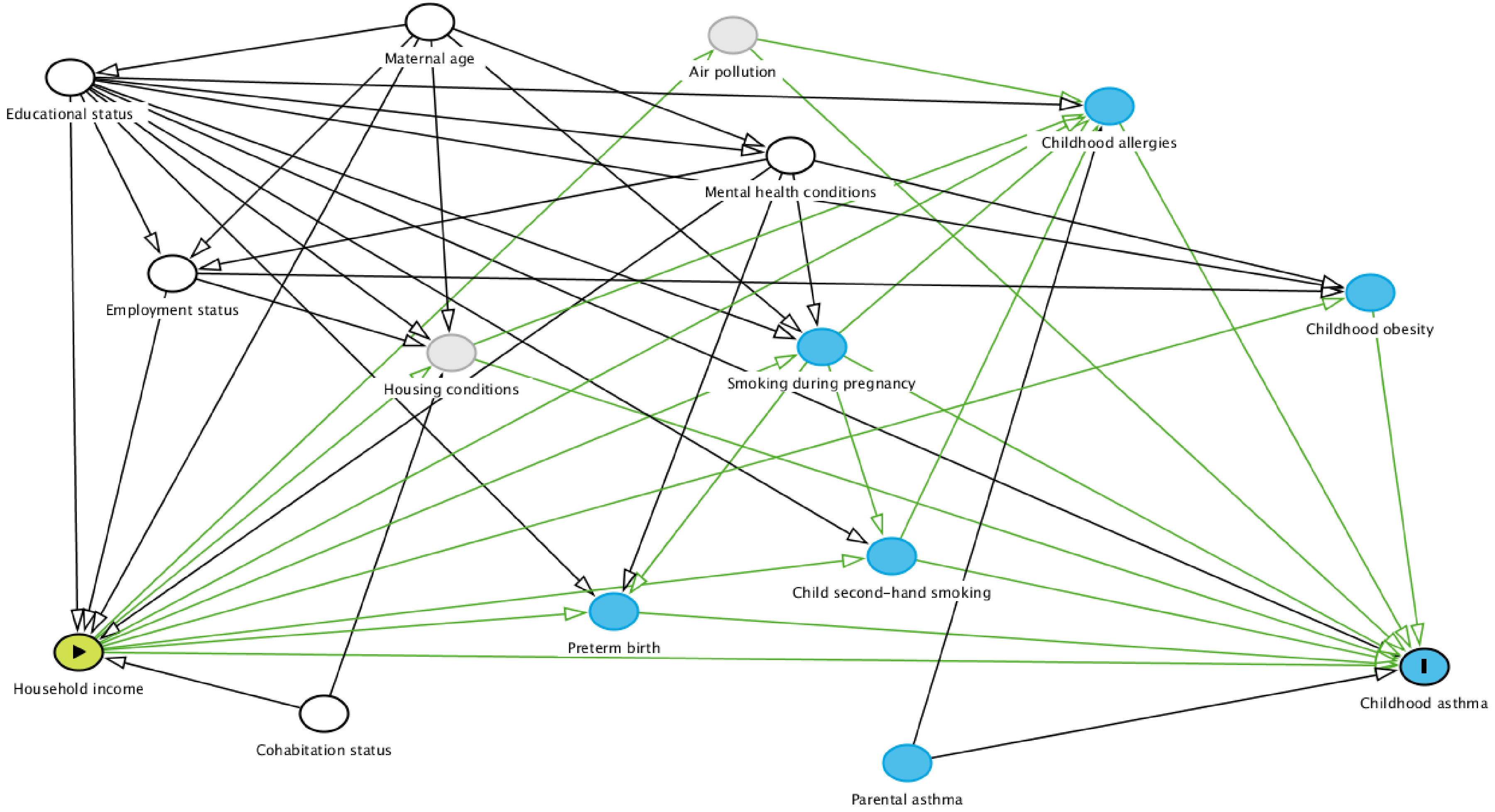

The white circles are the variables which are adjusted for. Green circle with arrow in the middle is the exposure of interest. The blue circle with a line in the middle is the outcome of interest. Grey circles are unobserved and blue circles are "ancestors of the outcome".[5,6]
The model code is shown in the following page.

DAG Model code:

dag {"Air pollution" [latent,pos="-0.650,-1.672"] "Child second-hand smoking" [pos="-0.291,0.110"] "Childhood allergies" [pos="0.202,-1.429"] "Childhood asthma" [outcome,pos="0.915,0.489"] "Childhood obesity" [pos="0.792,-0.791"] "Cohabitation status" [adjusted,pos="-1.576,0.649"] "Educational status" [adjusted,pos="-2.150,-1.528"] "Employment status" [adjusted,pos="-1.919,-0.857"] "Household income" [exposure,pos="-2.131,0.439"] "Housing conditions" [latent,pos="-1.287,-0.585"] "Maternal age" [adjusted,pos="-1.336,-1.717"] "Mental health conditions" [adjusted,pos="-0.520,-1.260"] "Parental asthma" [pos="-0.256,0.818"] "Preterm birth" [pos="-0.920,0.300"] "Smoking during pregnancy" [pos="-0.449,-0.606"] "Air pollution" -> "Childhood allergies" "Air pollution" -> "Childhood asthma" "Child second-hand smoking" -> "Childhood allergies" "Child second-hand smoking" -> "Childhood asthma" "Childhood allergies" -> "Childhood asthma" "Childhood obesity" -> "Childhood asthma" "Cohabitation status" -> "Household income" "Cohabitation status" -> "Housing conditions" "Educational status" -> "Child second-hand smoking" "Educational status" -> "Childhood allergies" "Educational status" -> "Childhood asthma" "Educational status" -> "Childhood obesity" "Educational status" -> "Employment status" "Educational status" -> "Household income" "Educational status" -> "Housing conditions" "Educational status" -> "Mental health conditions" "Educational status" -> "Preterm birth" "Educational status" -> "Smoking during pregnancy" "Employment status" -> "Childhood obesity" "Employment status" -> "Household income" "Employment status" -> "Housing conditions" "Household income" -> "Air pollution" "Household income" -> "Child second-hand smoking" "Household income" -> "Childhood allergies" "Household income" -> "Childhood asthma" "Household income" -> "Childhood obesity" "Household income" -> "Housing conditions" "Household income" -> "Preterm birth" "Household income" -> "Smoking during pregnancy" "Housing conditions" -> "Childhood allergies" "Housing conditions" -> "Childhood asthma" "Maternal age" -> "Educational status" "Maternal age" -> "Employment status" "Maternal age" -> "Household income" "Maternal age" -> "Housing conditions" "Maternal age" -> "Mental health conditions" "Maternal age" -> "Smoking during pregnancy" "Mental health conditions" -> "Childhood obesity" "Mental health conditions" -> "Employment status" "Mental health conditions" -> "Household income" "Mental health conditions" -> "Preterm birth" "Mental health conditions" -> "Smoking during pregnancy" "Parental asthma" -> "Childhood allergies" "Parental asthma" -> "Childhood asthma" "Preterm birth" -> "Childhood asthma" "Smoking during pregnancy" -> "Child second-hand smoking" "Smoking during pregnancy" -> "Childhood allergies" "Smoking during pregnancy" -> "Childhood asthma" "Smoking during pregnancy" -> "Preterm birth"}

DAGitty v3.0

Supplementary Table s3: Crude and adjusted risk ratio (RR) of more than 2 redeemed asthma prescriptions based on household income quartiles.

**Household Income**	*Medication* N (%)	**Crude RR (95% CI)**		**Adjusted RR (95% CI)**	
**< 25% (Q_1)**	13062 (7.1)	1.05	(1.03-1.08)	0.95	(0.92-0.98)
**< 25%, students (Q_{1e})**	1707 (6.9)	1.03	(0.97-1.08)	0.89	(0.84-0.94)
**25-50% (Q_2)**	16264 (7.8)	1.16	(1.13-1.18)	1.07	(1.04-1.09)
**50-75% (Q_3)**	15904 (7.6)	1.13	(1.10-1.15)	1.08	(1.06-1.11)
**>75% (Q_4)**	14126 (6.8)	*1.00*	*(ref)*	*1.00*	*(ref)*

Analysis adjusted for maternal mental health conditions, maternal education, employment status, cohabitation status, maternal age, and maternal country of origin.
CI: confidence interval

Supplementary Table s4: Crude and adjusted risk ratio (RR) of 1 asthma related hospital diagnosis based on household income quartiles

**Household Income**	*Hospitalization* N (%)	**Crude RR (95% CI)**		**Adjusted RR (95% CI)**	
**< 25% (Q_1)**	3774 (2.1)	1.43	(1.38-1.48)	1.18	(1.14-1.23)
**< 25%, students (Q_{1e})**	445 (1.8)	1.26	(1.17-1.35)	1.00	(0.93-1.07)
**25-50% (Q_2)**	3930 (1.9)	1.32	(1.28-1.37)	1.20	(1.16-1.24)
**50-75% (Q_3)**	3547 (1.7)	1.19	(1.15-1.23)	1.14	(1.10-1.18)
**>75% (Q_4)**	3009 (1.4)	*1.00*	*(ref)*	*1.00*	*(ref)*

Analysis adjusted for maternal mental health conditions, maternal education, employment status, cohabitation status, maternal age, and maternal country of origin.
CI: confidence interval

Supplementary Table s5: Adjusted risk ratio (RR) of more than 2 redeemed asthma prescriptions based on household income quartiles – including covariates from the father.

Medication

Household Income	Adjusted RR (95% CI)	
**< 25% (Q_1)**	0.94	(0.91-0.96)
**< 25%, students (Q_{1e})**	0.88	(0.84-0.93)
**25-50% (Q_2)**	1.05	(1.02-1.07)
**50-75% (Q_3)**	1.07	(1.05-1.10)
**>75% (Q_4)**	*1.00*	*(ref)*

Analysis adjusted for maternal and paternal mental health conditions, maternal and paternal education, maternal and paternal employment status, cohabitation status of both parents, maternal and paternal age, and maternal and paternal country of origin.
CI: confidence interval

Note that the study population in this analysis only consist of ***751,217 children*** *due to missing information on the paternal variables included in the adjustments.*

Supplementary Table s6: Adjusted risk ratio (RR) of 1 asthma related hospital diagnosis based on household income quartiles – including covariates from the father.

Hospitalization

Household Income	Adjusted RR (95% CI)	
**< 25% (Q_1)**	1.14	(1.09-1.19)
**< 25%, students (Q_{1e})**	0.98	(0.91-1.06)
**25-50% (Q_2)**	1.17	(1.13-1.21)
**50-75% (Q_3)**	1.12	(1.08-1.16)
**>75% (Q_4)**	*1.00*	*(ref)*

Analysis adjusted for maternal and paternal mental health conditions, maternal and paternal education, maternal and paternal employment status, cohabitation status of both parents, maternal and paternal age, and maternal and paternal country of origin.
CI: confidence interval

Note that the study population in this analysis only consist of ***751,217 children*** *due to missing information on the paternal variables included in the adjustments.*

References

1 Pottegård A, Schmidt SAJ, Wallach-Kildemoes H, Sørensen HT, Hallas J, Schmidt M. Data resource profile: The Danish national prescription registry. *Int J Epidemiol* 2017;46:798.

2 Sahl Andersen J, De Fine Olivarius N, Krasnik A. The Danish national health service register. *Scand J Public Health* 2011;39:34–37.

3 Schmidt M, Schmidt SAJ, Sandegaard JL, Ehrenstein V, Pedersen L, Sørensen HT. The Danish National patient registry: A review of content, data quality, and research potential. *Clin Epidemiol* 2015;7:449–90.

4 Jensen VM, Rasmussen AW. Danish education registers. *Scand J Public Health* 2011;39:91–94.

5 Greenland S, Pearl J, Robins J. Causal diagrams for epidemiologic research. *Epidemiology* 1999;10:37–48.

6 Howards PP, Schisterman EF, Poole C, Kaufman JS, Weinberg CR. 'Toward a clearer definition of confounding' revisited with directed acyclic graphs. *Am J Epidemiol* 2012;176:506–11.
